# Prooxidative Potential of Photo-Irradiated Aqueous Extracts of Grape Pomace, a Recyclable Resource from Winemaking Process

**DOI:** 10.1371/journal.pone.0158197

**Published:** 2016-06-24

**Authors:** Mana Tsukada, Takuji Nakashima, Toshiaki Kamachi, Yoshimi Niwano

**Affiliations:** 1 Graduate School of Bioscience and Biotechnology, Tokyo Institute of Technology, 2-12-1-M6-7 Ookayama, Meguro-ku, Tokyo, 152–8250, Japan; 2 Kitasato Institute for Life Sciences, Kitasato University, 5-9-1 Shirokane, Minato-ku, Tokyo, 108–8641, Japan; 3 School of Life Science and Technology, Tokyo Institute of Technology, 2-12-1-M6-7, Ookayama, Meguro-ku, Tokyo, 152–8250, Japan; 4 Tohoku University Graduate School of Dentistry, 4–1 Seiryo, Aoba-ku, Sendai, 980–8575, Japan; 5 HABA Laboratories Inc., 1-24-11, Kanda Sudacho, Chiyoda-ku, Tokyo, 101–0041, Japan; National Taiwan University, TAIWAN

## Abstract

Our previous study revealed that aqueous extract of grape pomace obtained from a winemaking process could exert bactericidal action upon photo-irradiation via reactive oxygen species (ROS) formation. In the present study, we focused on chemical composition and prooxidative profile of the extract. Liquid chromatography-electrospray ionization-mass spectrometry (LC-ESI-MS) analysis showed that polyphenolic compounds including catechin monomers, dimers, trimers, and polyphenolic glucosides were contained. The polyphenol rich fraction used for the LC-ESI-MS analysis generated hydrogen peroxide (H_2_O_2_) upon photo-irradiation possibly initiated by photo-oxidation of phenolic hydroxyl group. That is, reduction of dissolved oxygen by proton-coupled electron transferred from the photo-oxidized phenolic hydroxyl group would form H_2_O_2_. The resultant H_2_O_2_ was then photolyzed to generate hydroxyl radical (•OH). The prooxidative profile of the extract in terms of •OH generation pattern upon photo-irradiation was similar to that of grape seed extract (GSE) as an authentic polyphenol product and (+)-catechin as a pure polyphenolic compound, and in all the three samples •OH generation could be retained during photo-irradiation for at least a couple of hours. The prooxidant activity of the photo-irradiated extract indicated by •OH yield was more potent than that of the photo-irradiated GSE and (+)-catechin, and this was well reflected in their bactericidal activity in which the photo-irradiated extract could kill the bacteria more efficiently than did the photo-irradiated GSE and (+)-catechin.

## Introduction

Grape is the largest fruit crop in the world. The annual production worldwide amounts to almost 70 million tons and around 80% is used to make wine [1], indicating that waste materials or byproducts obtained from winemaking process could be a valuable resource to be recycled. The waste from winemaking process can be divided into three categories, *i*.*e*., pomace, clarification sediment such as lees, and yeast sediment. Regarding grape pomace, for instance, it was reported that over 16 million tons of byproducts were produced [2]. As one sphere of beneficial use of waste materials or byproducts obtained from winemaking process, a recent study showed that grape pomace appeared to have the great potential as source of natural antioxidant and antimicrobial agents [3].

Our previous study revealed that photo-irradiation of the aqueous extract from grape pomace obtained from a winemaking process exerted bactericidal effect on *Staphylococcus aureus* due to highly reactive hydroxyl radial (•OH) formation [4]. The grape pomace obtained from a winemaking process could be a substantial resource of polyphenolic compounds [5]. Since it has been reported that some polyphenolic compounds such as gallic acid, caffeic acid, chlorogenic acid, and proanthocyanidin exerts bactericidal activity upon photo-irradiation [6–8], it is speculated that polyphenolic compounds in the aqueous extract from grape pomace would be responsible for the bactericidal activity upon photo-irradiation.

The purpose of the present study was to assess the chemical composition in the aqueous extracts of grape pomace by liquid chromatography-electrospray ionization-mass spectrometry (LC-ESI-MS). In addition, prooxidative profile and potential indicated by •OH generation induced by photo-irradiation were compared to those of commercially available grape seed extract as an authentic polyphenol product and (+)-catechin as a pure polyphenolic compound.

## Materials and Methods

### Reagent

Reagents were purchased from the following sources: 5,5-dimethyl-1-pyrroline *N*-oxide (DMPO) from Labotec (Tokyo, Japan); catalase (from bovine liver) from Wako Pure Chemical Industries (Osaka, Japan); 4-hydroxy-2,2,6,6-tetramethylpiperidine *N*-oxyl (TEMPOL) from Sigma Aldrich (St. Louis, MO), (+)-catechin from Tokyo Chemical Industry (Tokyo, Japan). GSE (Leucoselect^®^) was obtained from Indena (Milan, Italy). According to the manufacture, Leucoselect^®^ is a grape seed extract with a well-defined chemical composition such as catechin-monomers and -oligomers, which was completely elucidated by instrumental analyses such as high performance LC-MS. This was also confirmed in a previous study in which Leucoselect^®^ was subjected to a LC-MS analysis showing that the calculated concentrations of (+)-catechin and (–)-epicatechin were 12.1% (wt/wt) and 6.6% (wt/wt), respectively [9]. All other reagents used were of analytical grade.

### Preparation of aqueous extract

According to our previous study [4], aqueous extract was prepared from residue of crushed grape (hereafter termed as grape pomace). A grape variety Niagara harvested in Hokkaido, Japan was subjected to a vinification process of white wine. The grape pomace obtained from crushed and pressed fruitage (including peel and seed) was freeze-dried. Three times volume of pure water (at the ratio of 3 ml pure water per 1 g powder) was added to each dried powder and the resultant mixture was agitated at 150 rpm overnight at room temperature. The upper layer was taken and centrifuged at 3000 rpm for 20 min to obtain supernatant. Following membrane filtration (φ0.22 μm) the supernatant was subjected to total polyphenol determination by Folin-Denis method in which gallic acid was used as a standard [10]. The aqueous extract solution (hereafter termed as GPE for grape pomace extract) was stored at -20°C until assayed. In use, GPE was adjusted to contain designated concentrations of total polyphenol with pure water.

### Light source

According to our previous studies [4,6], an experimental device equipped with a light emitting diode with a wavelength of 400 nm (NHH105UV, Lustrous Technology, Shiji, Taiwan) was used. The output power of the LED measured using a power meter (FieldMate, Coherent, Santa Clara, CA) was set to be 400 mW per LED corresponding to an irradiance of 130 mW/cm^2^ at a distance of 15 mm from the LED. A four-clear-sided methacrylate plastic cuvette containing the sample was placed in the experimental device. LED-light irradiation was performed toward both sides of the plastic cuvette (total irradiance: 260 mW/cm^2^).

### LC-ESI-MS analysis for chemical composition of GPE

For LC-ESI-MS analysis, since water soluble fraction was not suitable for MS analysis because of apprehensiveness of contamination in the ionization chamber, a direct analysis of GPE could not be carried out. Instead, 100 ml of GPE was concentrated to dryness *in vacuo*, and extracted twice with 10 ml of methanol (MeOH) followed by centrifugation at 9, 000 × g for 10 min. The supernatant was further concentrated to dryness *in vacuo*, and the resultant dried residue was dissolved in MeOH to be 100 mg/ml followed by passage through a filter (polyvinylidene difluoride; pore size, 0.2 μm). The resultant sample was injected into the electrospray ion source of a QSTAR Elite ESI quadruple time-of-flight mass spectrometer (AB Sciex; Framingham, MA, USA) coupled to Agilent 1200 series (Agilent Technologies, Santa Clara, CA, USA). Chromatographic separation was undertaken on an Inertsil ODS-4 (3.0 × 250 mm, GL Sciences, Tokyo, Japan) at 40°C. With regard to gradient elution, solvent A was water with 2 mM ammonium acetate, and B was methanol with 2 mM ammonium acetate. Gradient elution was 0–30 min and 5–100% B. Flow rate was 0.5 ml/min, the injection volume was 5 μl, and UV detection was carried out by a photodiode array detector. ESI-MS was recorded for 30 min in the *m/z* region from 100 to 2000 Da with the following instrument parameters: ion spray voltage = 5500 V, source gas = 50 l/min, curtain gas = 30 l/min, declustering potential = 50V, focusing potential = 250 V, temperature = 450°C, and detector voltage = 2300 V. LC-MS analysis was undertaken by high-resolution ESI-MS (R ≥ 10,000; tolerance for mass accuracy = 5 ppm). As standards, (+)-catechin (Tokyo Chemical Industry, Tokyo, Japan) and (–)-epicatechin (Sigma-Aldrich) were used.

### Total polyphenol determination of MeOH soluble and insoluble fractions of GPE

Since LC-ESI-MS analysis as described above revealed that phenolic compounds were contained in MeOH soluble fraction, total polyphenol concentrations of MeOH soluble and insoluble fractions of GPE were compared. An aliquot (50 ml) of GPE was concentrated to dryness *in vacuo*, and extracted twice with 10 ml of MeOH followed by centrifugation at 8, 000 rpm for 10 min to obtain supernatant and precipitate. The supernatant was further concentrated to dryness *in vacuo*, and the resultant dried residue was reconstituted in pure water to be 20 ml as a MeOH soluble fraction. The precipitate obtained after centrifugation was reconstituted in pure water to be 20 ml as a MeOH insoluble fraction. Total polyphenol concentrations in the two fractions were determined by Folin-Denis method as described above.

### ROS generation of photo-irradiated MeOH soluble and insoluble fractions of GPE

Hydrogen peroxide (H_2_O_2_) and •OH generation of photo-irradiated MeOH soluble and insoluble fractions of GPE were examined as described in our previous study [4]. For H_2_O_2_ determination, 500 μl each of the MeOH soluble and insoluble fractions placed in a plastic cuvette was irradiated with LED light for 0, 5, and 10 min. Immediately after the irradiation, H_2_O_2_ concentration was determined by a colorimetric method based on the peroxide-mediated oxidation of Fe^2+^ followed by the reaction of Fe^3+^ with xylenol orange [11]. For •OH determination in photo-irradiated fractions was performed using an ESR spin trapping technique. An aliquot (483 μl) of the sample was mixed with 17 μl of DMPO in a plastic cuvette to reach a final concentration of 300 mM for DMPO. Then, the mixed sample was irradiated with LED light for 15, 30, and 60 s. For a negative control, each mixed sample kept in a light-shielding box for 60 s was subjected to ESR determination of •OH. After irradiation, the sample was transferred to a quartz cell for ESR spectrometry, and the ESR spectrum was recorded on an X-band ESR spectrometer (JES-FA-100; JEOL, Tokyo, Japan). The measurement conditions for ESR were as follows: field sweep, 331.89–341.89 mT; field modulation frequency, 100 kHz; field modulation width, 0.1 mT; amplitude, 200; sweep time, 2 min; time constant, 0.03 s; microwave frequency, 9.420 GHz; and microwave power, 4 mW. TEMPOL (2 μM) was used as a standard to calculate the concentration of spin-trapped radicals, and the ESR spectrum of manganese (Mn^2+^) held in the ESR cavity was used as an internal standard. All tests were performed in triplicate.

### Comparison of •OH generation in photo-irradiated GPE and polyphenols

Firstly, concentration effect of GPE, GSE, and (+)-catechin on DMPO-OH (a spin adduct of DMPO and •OH) generation was examined. GPE was diluted with pure water to contain designated concentrations of total polyphenol. Both GSE and (+)-catechin were dissolved in pure water to make designated concentrations. An aliquot (483 μl) of the sample was mixed with 17 μl of DMPO in a plastic cuvette to reach a final concentration of 300 mM for DMPO. Then, the mixed sample was irradiated with LED light for 1 min followed by the ERS analysis as described above.

Secondly, photo-irradiation time effect on DMPO-OH formation was examined. GPE, GSE, and (+)-catechin samples were mixed with DMPO as described above (final concentrations of GPE, GSE, (+)-catechin, and DMPO were 0.25 mg total polyphenol, 1 mg, 1 mg, and 300 mM, respectively), and was subjected to LED-light irradiation for designated time period followed by the ESR analysis as described above.

Thirdly, since irradiation time effect experiment as described above revealed that DMPO-OH level in all tested samples reached a plateau at irradiation time of around 1 min, it was examined if •OH was continuously generated during LED-light irradiation for longer than 1 min. In this experiment, DMPO was added to GPE, GSE, and (+)-catechin samples irradiated in advance with LED light for up to 2 hr (final concentrations of GPE, GSE, (+)-catechin, and DMPO were 0.25 mg total polyphenol, 1 mg, 1 mg, and 300 mM, respectively). Immediately after addition of DMPO, the sample was further irradiated with LED light for 1 min. Then ESR analysis was performed as described above.

Finally, to examine why DMPO-OH level reached a plateau, the following two experiments were conducted. One was examination of decay of DMPO-OH after LED-light irradiation for 1 min. GPE, GSE and (+)-catechin samples were irradiated with LED light for 1 min in the presence of DMPO as described above, and then time course changes in DMPO-OH level were monitored under a light shielding condition for up to 10 min. The other was examination of DMPO-OH generation under following there conditions; 1) each sample with 300 mM DMPO was irradiated with LED light for 1 min, and kept under the light shielding condition for 1 min, 2) each sample with 300 mM DMPO was irradiated with LED light for 2 min, and 3) each sample without DMPO was irradiated with LED light for 1 min, and further irradiated in the presence of 300 mM DMPO for 1 min. Then ESR analysis was performed as described above. In the two experiments, final concentrations of GPE, GSE, and (+)-catechin were 0.25 mg total polyphenol, 1 mg, and 1 mg, respectively. All tests were performed in triplicate.

### Bactericidal assay

*Staphylococcus aureus* JCM 2413 purchased from the Japan Collection of Microorganisms, RIKEN BioResource Center (Wako, Japan) was used. A bacterial suspension was prepared in sterile physiological saline from a culture grown on brain heart infusion (BHI) agar (Becton Dickinson Labware, Franklin Lakes, NJ) aerobically at 37°C overnight. In a plastic cuvette, 483 μl of sample was mixed with 17 μl of the bacterial suspension to reach final concentration of approximately 10^7^ colony forming units (CFU)/ml for the bacteria. Then, the samples were exposed to LED light for 10 min. After irradiation, 50 μl of the sample was mixed with an equal volume of sterile catalase solution (5000 U/ml phosphate buffered saline (pH 7.4)) to eliminate the effect of generated H_2_O_2_. A 10-fold serial dilution of the mixture was prepared using sterile physiological saline, and 10 μl of the diluted solution was seeded onto a BHI agar plate. The agar plates were cultured in the same way as described above for 2 days, and the CFU/ml was determined. In addition, each sample with the bacterial suspension that was kept for 10 min under a light shielding condition instead of being exposed to LED light was subjected to the same procedure. All tests were performed in triplicate.

### Scavenging activity against •OH generated by a Fenton reaction

The assay used in this study was essentially identical to that described in a previous paper [12]. In brief, 50 μl of 2 mM H_2_O_2_, 50 μl of 89 mM DMPO, 50 μl of sample, and 50 μl of 0.2 mM FeSO_4_ were placed in a test tube and mixed. Each mixture was transferred to an ESR spectrometry cell and the DMPO-OH spin adduct was quantified 60 s after the addition of FeSO_4_. The measurement conditions for ESR were essentially identical to those described above.

### Statistical analyses

Statistical analyses were undertaken using Tukey–Kramer multiple comparison test and Student’s *t* test for pairwise comparisons between multiple groups and comparison between two groups, respectively. Regarding the statistical significance in the CFU/ml obtained in the bactericidal assay assessed by Tukey-Kramer multi-comparison test, the analysis was performed following logarithmic conversion. Two-way analysis of variance (ANOVA) was also performed to determine if prior-irradiation time and post-irradiation time were significantly affected the DMPO-OH level. *P*<0.05 was considered significant.

## Results

### LC-ESI-MS analysis for chemical composition of GPE

**Fig 1:**
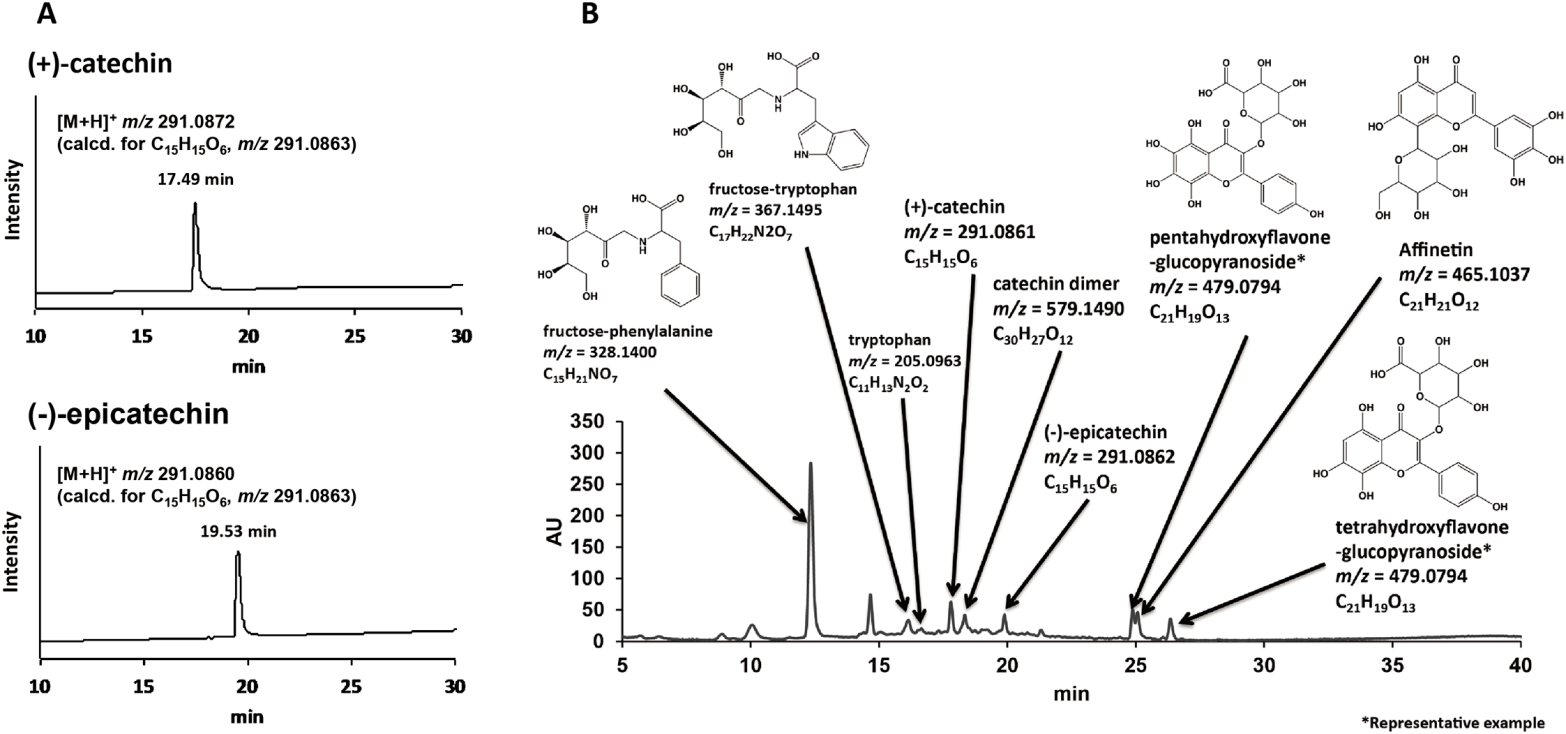
LC-ESI-MS analyses of standard reagents (A) and representative LC chromatogram with estimated chemical structural formulas obtained from MeOH soluble fraction of GPE (B).

**Fig 2:**
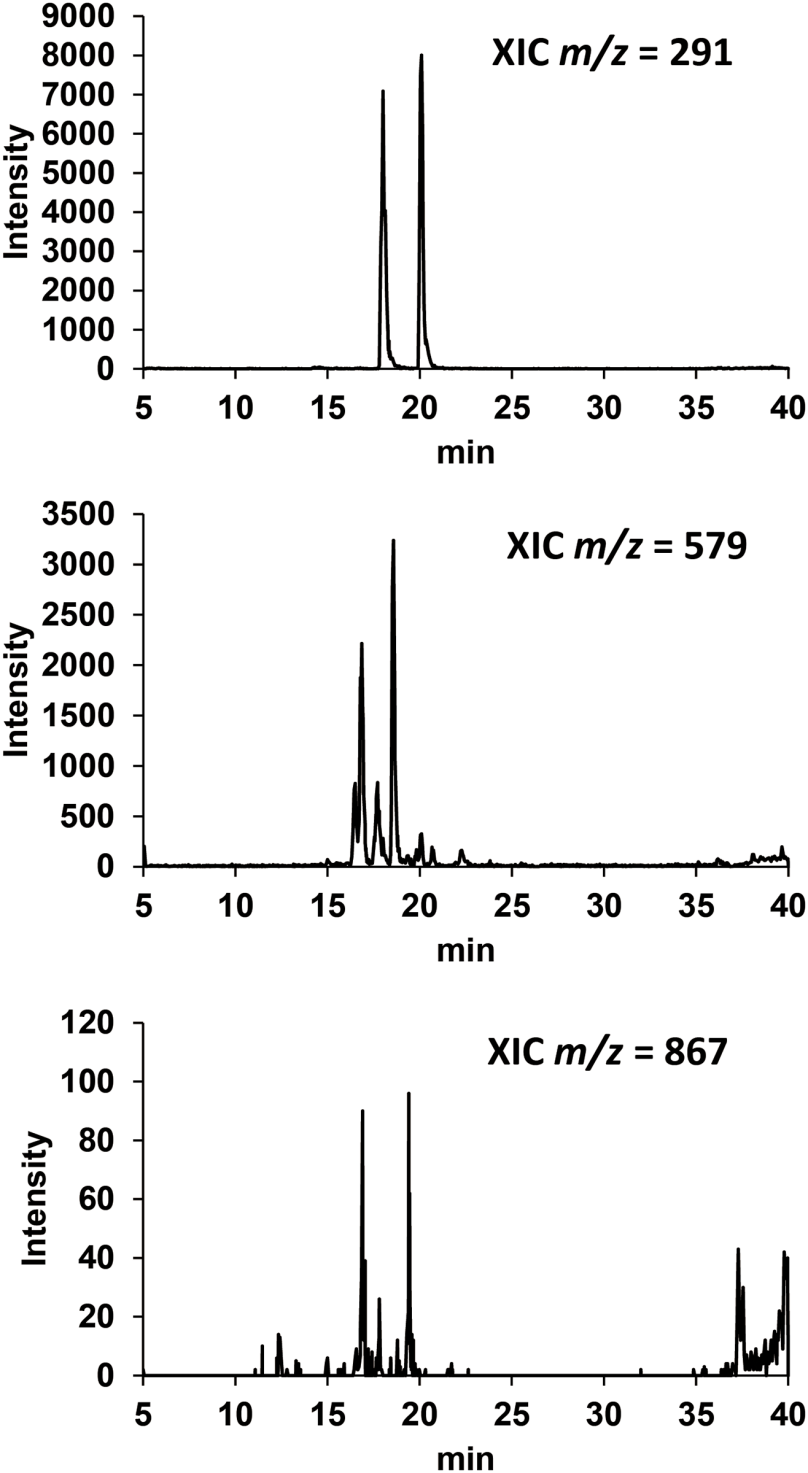
Extracted-ion chromatograms (XICs) with m/z corresponding to catechin monomers, dimers, and trimers.

The MS and UV spectra of each peak were compared with those of known compounds using existing databases, the Dictionary of Natural Products (http://dnp.chemnetbase.com/). A representative LC chromatogram with estimated chemical structural formulas obtained from MeOH soluble fraction of GPE is shown in Fig 1. The fraction contained phenolic compounds including (+)-catechin, (-)-epicatechin, catechin dimers, and polyphenolic glucosides. Fig 2 shows extracted ion chromatograms of *m/z* 291, 579, and 867, obtained from MeOH fraction of GPE, indicating that the fraction contained catechin monomers, dimers, and trimmers. Based on the assumption that catechin monomers would not be contained in MeOH insoluble fraction, the calculated concentrations of (+)-catechin and (–)-epicatechin in GPE with 0.5 mg/ml total polyphenol were approximately 20 and 8 μg/ml, respectively.

### Total polyphenol content of MeOH soluble and insoluble fractions of GPE, and ROS generation upon photo-irradiation

**Fig 3:**
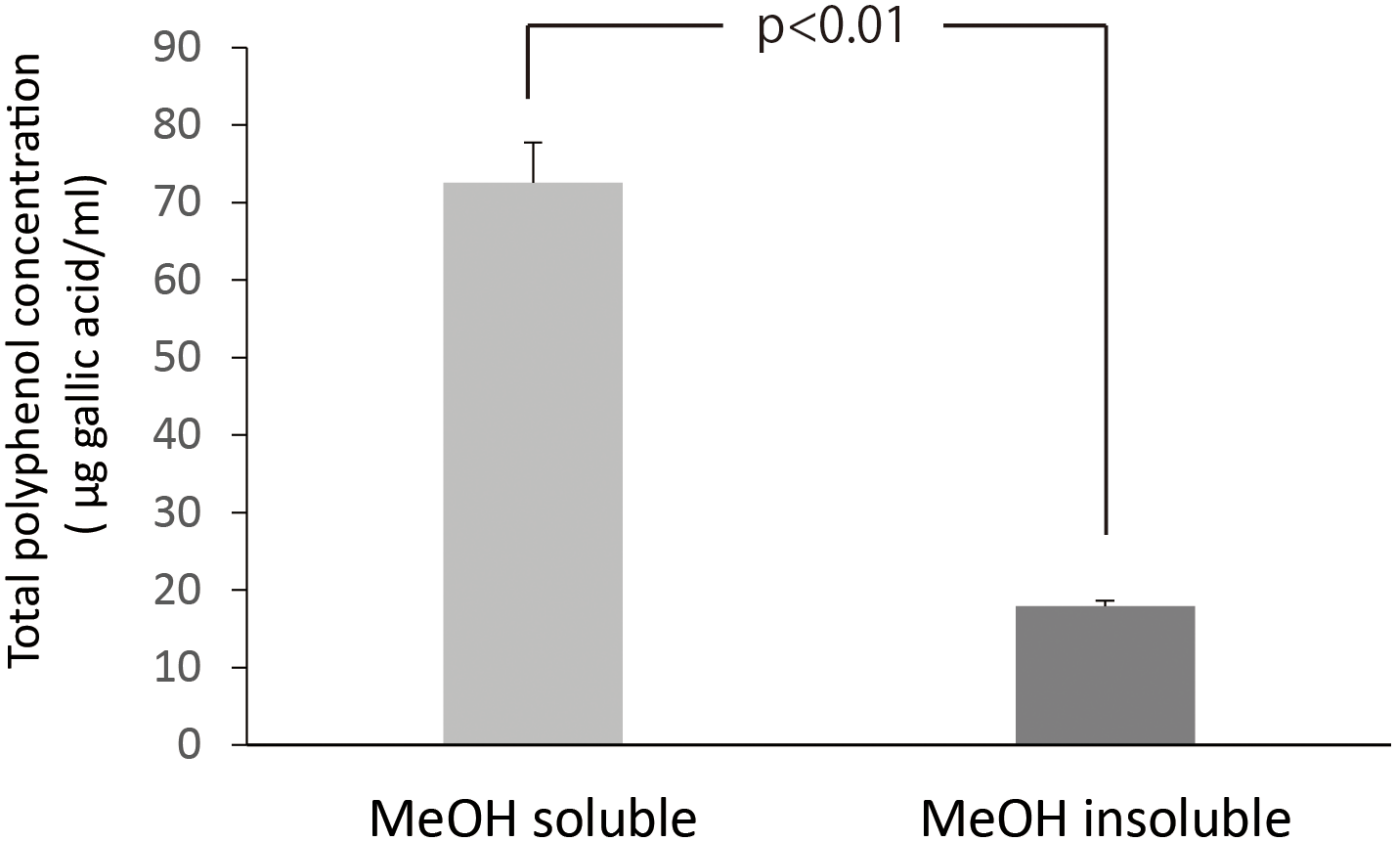
Total polyphenol concentrations in MeOH soluble and insoluble fractions. Each value represents the mean with standard deviation (n = 3).

**Fig 4:**
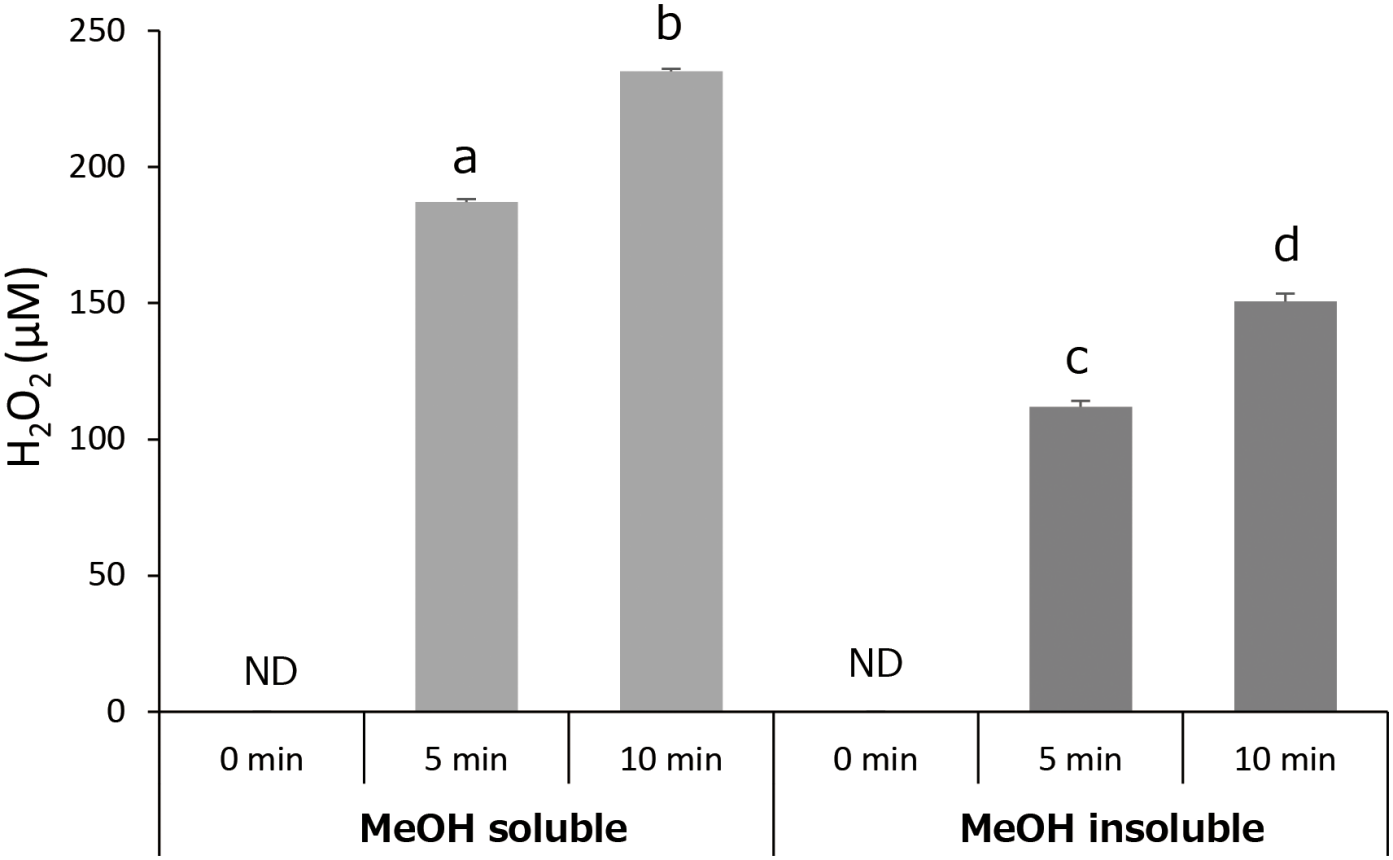
Effect of photo-irradiation time on the yield of H_2_O_2_ in MeOH soluble and insoluble fractions. Each value represents the mean with standard deviation (n = 3). Significant differences (*p*<0.05) are denoted by different alphabetical letters. ND: not detected.

**Fig 5:**
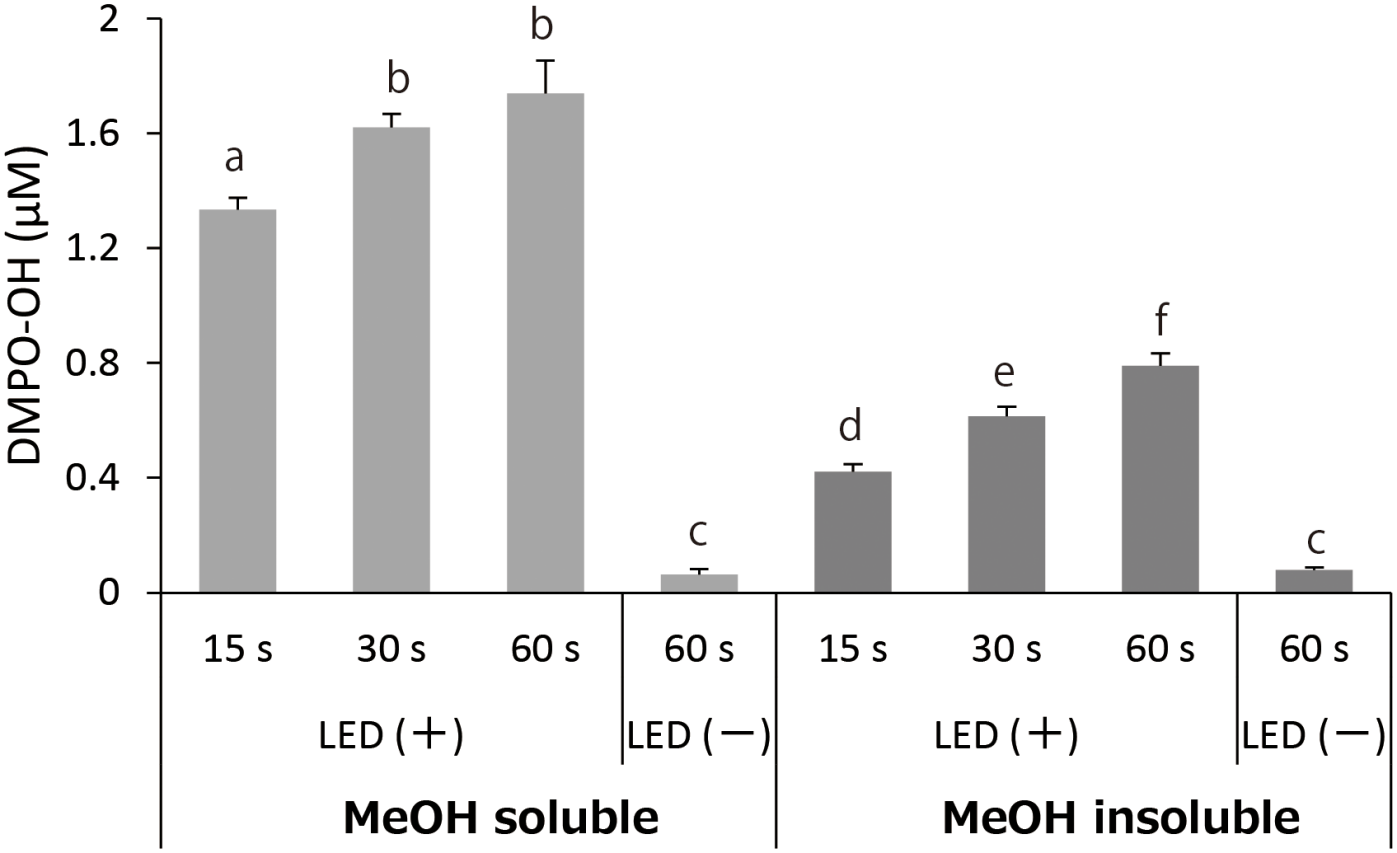
Effect of photo-irradiation time on the yield of •OH (expressed as DMPO-OH) in MeOH soluble and insoluble fractions. Each value represents the mean with standard deviation (n = 3). Significant differences (p<0.05) are denoted by different alphabetical letters. LED(-): Kept under a light shielding condition.

Total polyphenol contents of MeOH soluble and insoluble fractions are summarized in Fig 3. Total polyphenol content of MeOH soluble fraction was approximately 3.5 times higher than that of MeOH insoluble fraction (*p*<0.01). Fig 4 shows H_2_O_2_ concentrations in photo-irradiated MeOH soluble and insoluble fractions. Although H_2_O_2_ formation increased with irradiation time in the both fractions, the resultant H_2_O_2_ concentrations in MeOH fraction were much higher than those in corresponding MeOH insoluble fractions (*p*<0.05). Fig 5 summarizes the effect of photo-irradiation time on •OH generation in MeOH soluble and insoluble fractions. As was the case with H_2_O_2_, •OH generation as expressed as DMPO-OH concentration increased with irradiation time in the both fractions, and the resultant DMPO-OH concentrations in MeOH soluble fraction were much higher than those in corresponding MeOH insoluble fractions (*p*<0.05). DMPO-OH levels in the negative controls (kept under a light-shielding condition for 60 s) were trace levels in the both fractions.

### Comparison of •OH generation in photo-irradiated GPE and polyphenols

**Fig 6:**
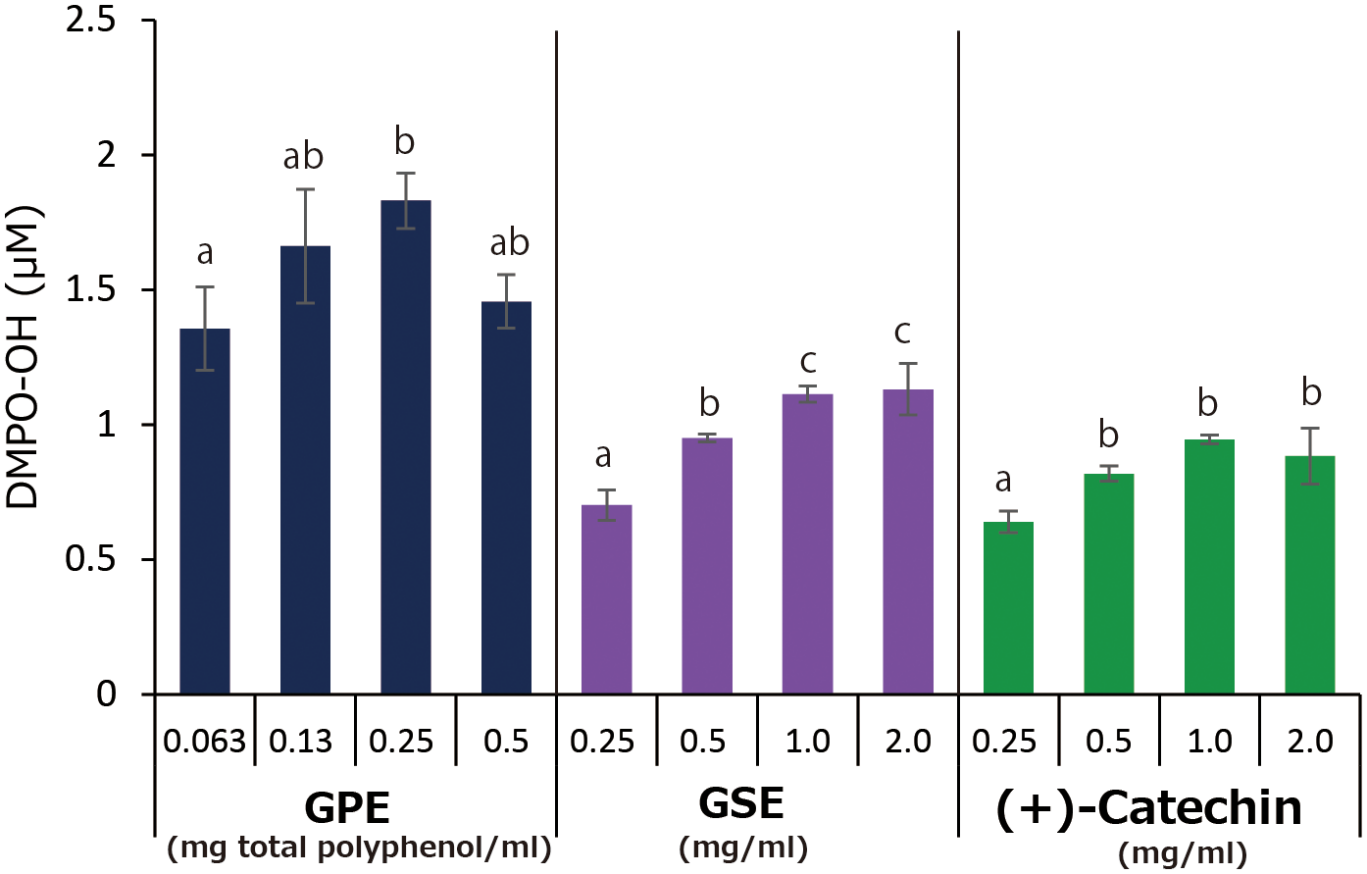
Concentration effect of GPE, GSE, and (+)-catechin on •OH (expressed as DMO-OH) generation during LED-light irradiation for 1 min. Each value represents the mean with standard deviation (n = 3). Significant differences (*p*<0.05) in each group are denoted by different alphabetical letters.

Concentration effect of GPE, GSE, and (+)-catechin on •OH generation during LED-light irradiation for 1 min is summarized in Fig 6. Very slight concentration dependency in DMPO-OH concentration was observed in GPE up to 0.25 mg/ml of total polyphenol, but no increase in the concentration was found in 0.5 mg/ml of total polyphenol. Similarly, DMPO-OH levels in photo-irradiated GSE and (+)-catechin samples increased slightly with concentrations of GSE and (+)-catechin up to 1.0 mg/ml, and no increases in DMPO-OH concentrations were found in 2.0 mg/ml of both GSE and (+)-catechin. In whole. DMPO-OH generation was in the order of GPE > GSE ≥ (+)-catechin.

**Fig 7:**
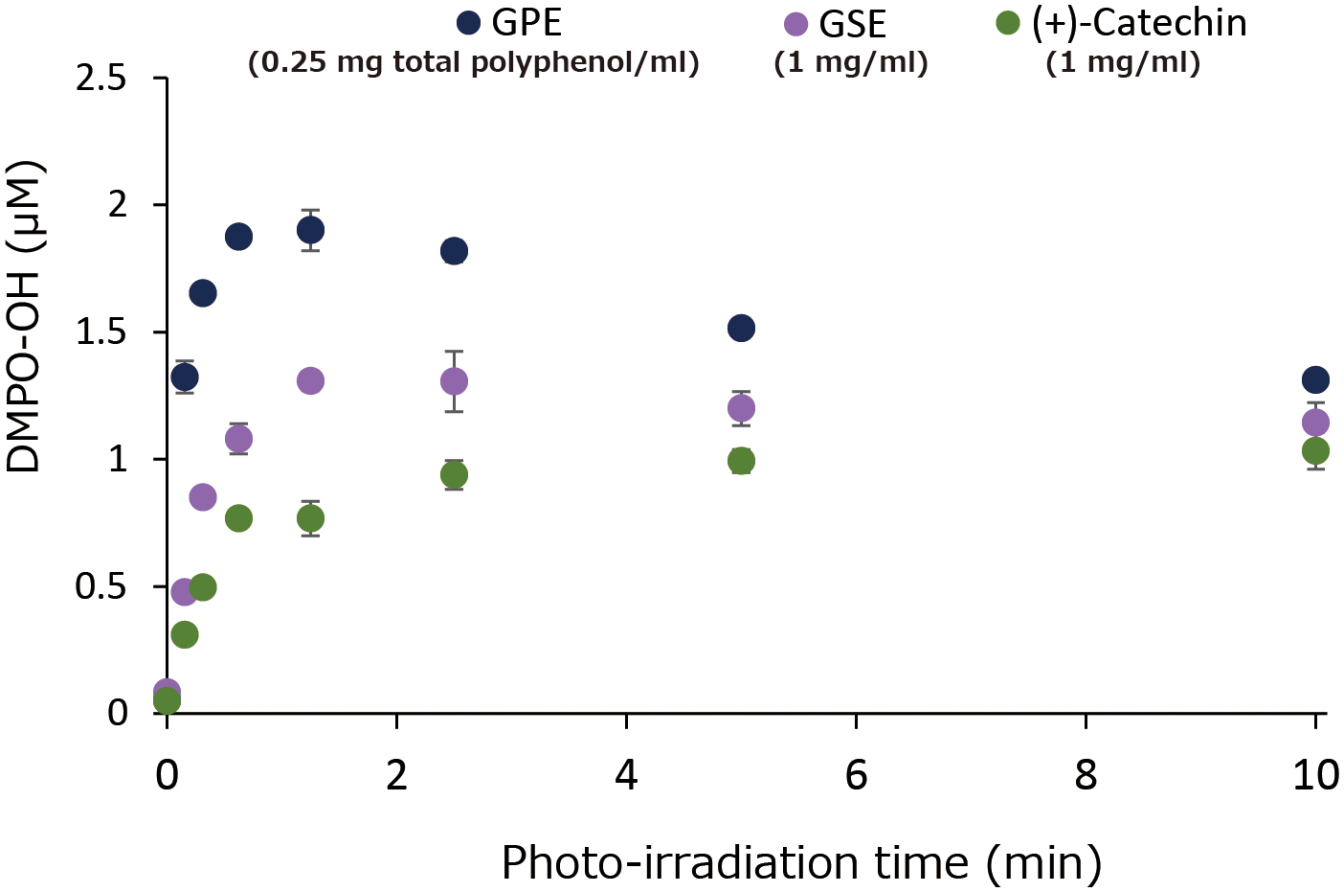
Effect of photo-irradiation time on DMPO-OH yields. Each value represents the mean with standard deviation (n = 3).

Fig 7 summarizes the photo-irradiation time effect on DMPO-OH generation. DMPO-OH levels in all the samples reached a plateau at around 1 min of irradiation. As was the case with Fig 5, DMPO-OH generation in whole was in the order of GPE > GSE ≥ (+)-catechin.

**Fig 8:**
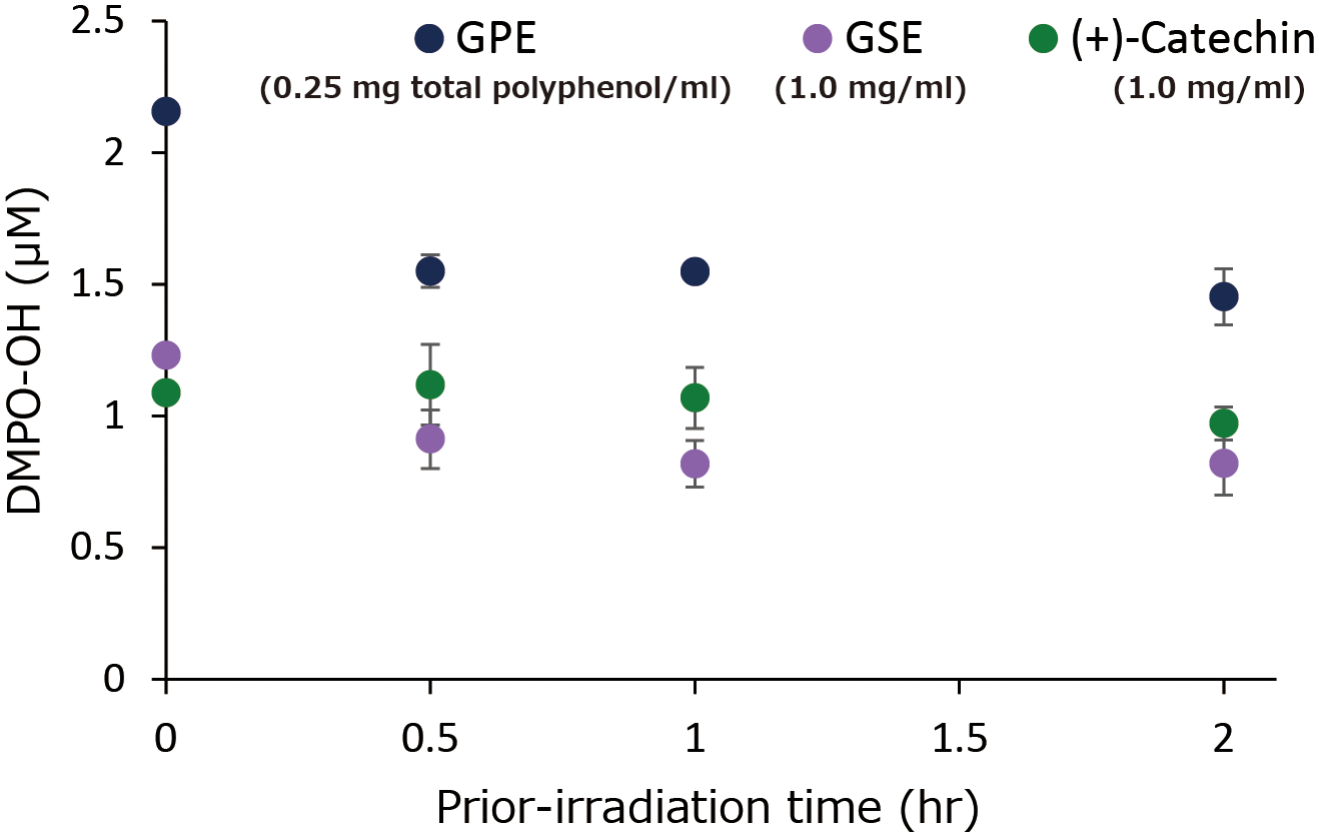
Effect of prior-irradiation time in the absence of DMPO on DMPO-OH yielded after additional LED-light irradiation for 1 min in the presence of DMPO. Each value represents the mean with standard deviation (n = 3).

Fig 8 shows the effect of time for prior-irradiation of each sample without DMPO on the DMPO-OH generation after additional LED-light irradiation of each sample with DMPO for 1 min, and the result of the two way ANOVA is summarized in Table 1. The two-way ANOVA showed a significant prior-irradiation time effect on DMPO-OH generation. Indeed, in GPE (0.25 mg/ml of total polyphenol) and GSE (1.0 mg/ml), slight decreases in DMPO-OH levels were observed with prior-irradiation time, and the levels reduced by around 30% in the both samples irradiated in advance for 2 hr as compared with those without prior-irradiation. DMPO-OH levels in (+)-catechin (1.0 mg/ml) did not significantly change, and almost the same level was kept even after irradiated in advance for 2 hr.

**Table 1:** Summary table of two-way ANOVA for the prior-irradiation time effect on DMPO-OH generation.

	df	Sum of squares	Mean square	F value	P value
Sample*	2	2.017	1.0087	137.678	<0.0001
Time	4	1.0997	0.275	37.5213	<0.0001
Sample x Time	8	0.495	0.062	8.4511	<0.0001
Error	30	0.220	0.007		

*GPE, GSE, and (+)-catechin

df: degree of freedom

**Fig 9:**
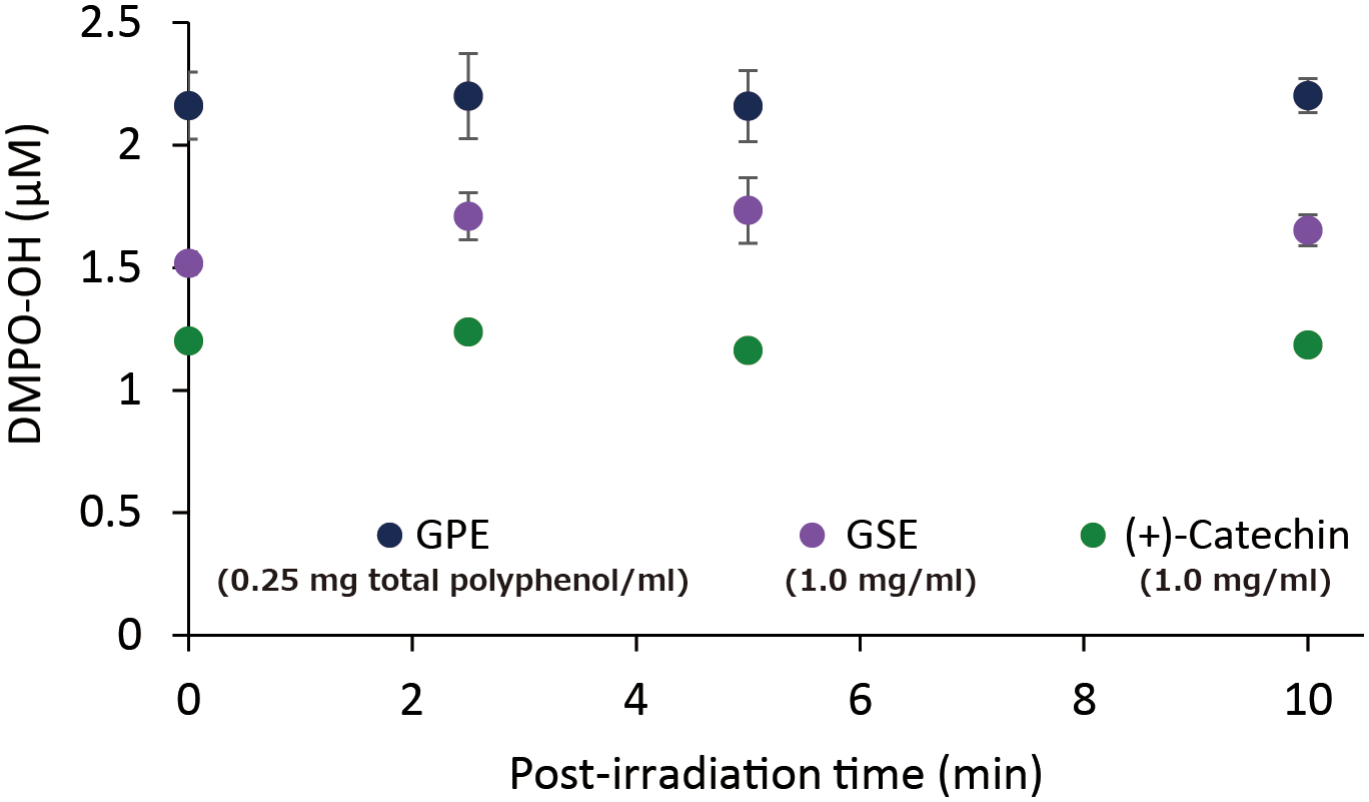
Time course changes in DMPO-OH level after termination of photo-irradiation. Each value represents the mean with standard deviation (n = 3).

Fig 9 summarizes time course changes in DMPO-OH level after termination of photo-irradiation, and the result of the two way ANOVA is summarized in Table 2. The two-way ANOVA showed no significant post-irradiation time effect on DMPO-OH levels. Indeed, DMPO-OH generated by photo-irradiation was relatively stable at least for 10 min regardless of the initial levels.

**Table 2:** Summary table of two-way ANOVA for the post-irradiation time effect on DMO-OH.

	df	Sum of squares	Mean square	F value	P value
Sample*	2	1.430	0.715	77.7652	<0.0001
Time	3	0.036	0.0125	1.3205	0.2909
Sample x Time	6	0.061	0.010	1.0992	0.3915
Error	24	0.221	0.009		

*GPE, GSE, and (+)-catechin

df: degree of freedom

**Fig 10:**
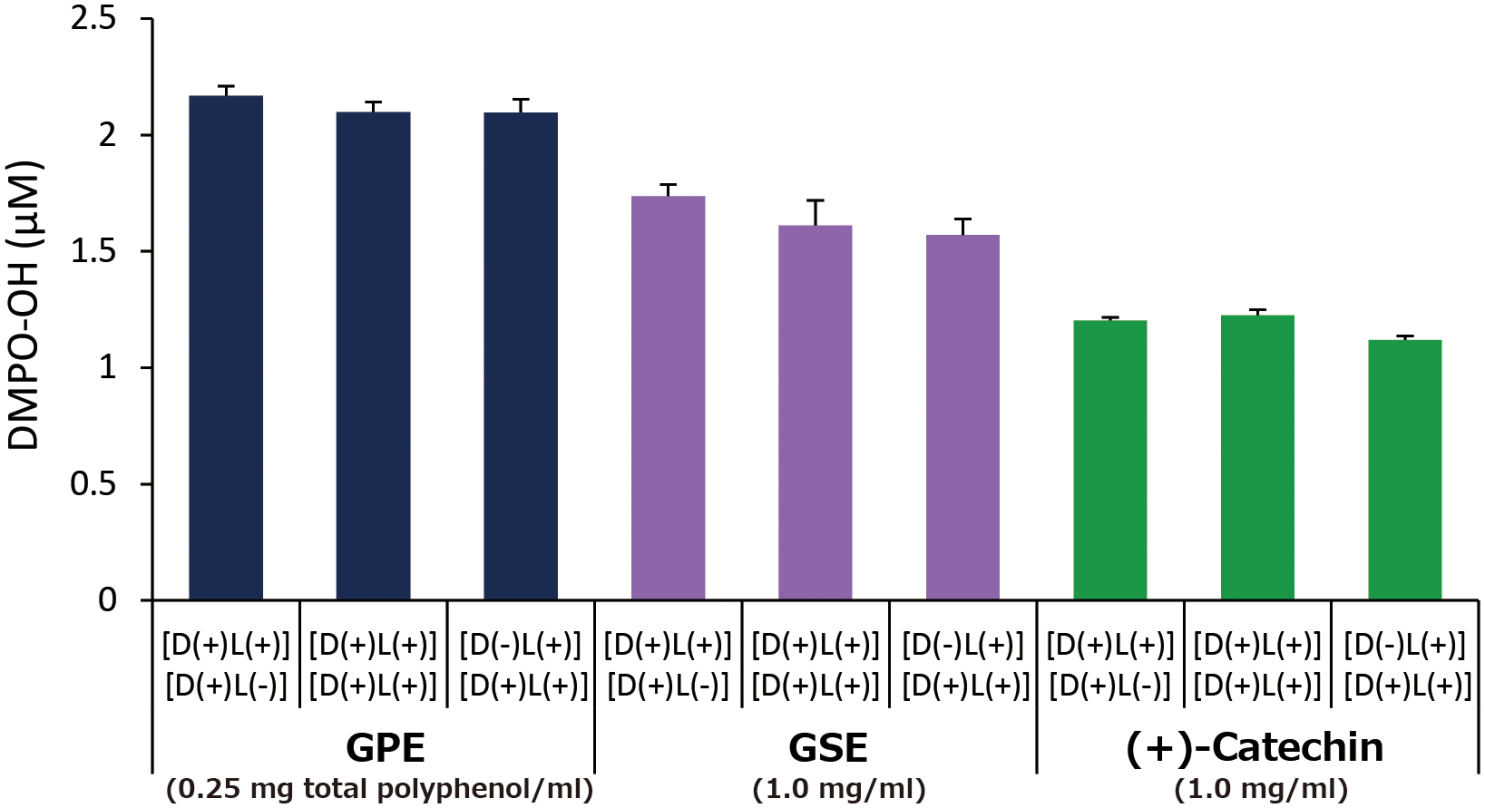
Effect of different treatment conditions on DMPO-OH yields. Each value represents the mean with standard deviation (n = 3). [D(+)L(+)][D(+)L(-)]: sample with DMPO was photo-irradiated for 1 min, and kept under a light shielding condition for 1 min, [D(+)L(+)][D(+)L(+)]: sample with DMPO was photo-irradiated for 2 min, and [D(-)L(+)][D(+)L(+)]: sample without DMPO was photo-irradiated for 1 min, and further irradiated with DMPO for 1 min.

Fig 10 shows DMPO-OH generation under following there conditions: 1) each sample with 300 mM DMPO was irradiated with LED light for 1 min, and kept under a light shielding condition for 1 min, expressed as [D(+)L(+)][D(+)L(-)], 2) each sample with 300 mM DMPO was irradiated with LED light for 2 min, expressed as [D(+)L(+)][D(+)L(+)], and 3) each sample without DMPO was irradiated with LED light for 1 min, and further irradiated in the presence of 300 mM DMPO for 1 min, expressed as [D(-)L(+)][D(+)L(+)], where D and L indicate DMPO and LED light, respectively. DMPO-OH levels in each sample were almost the same regardless of the L and D treatment conditions.

### Bactericidal assay

**Fig 11:**
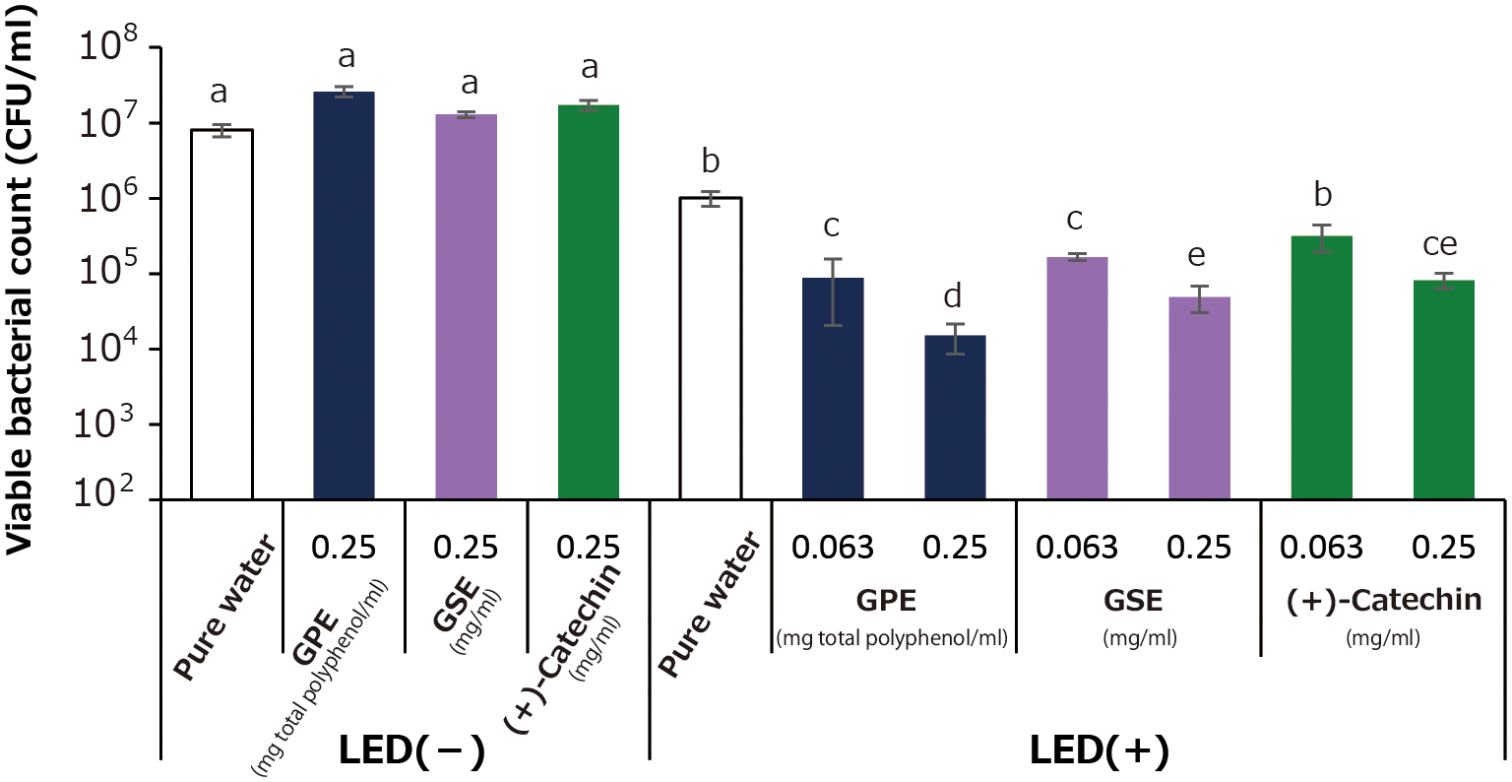
Bactericidal activity of photo-irradiated GPE, GSE, and (+)-catechin on *Staphylococcus aureus*. LED light was irradiated for 10 min. Each value represents the mean with standard deviation (n = 3). Significant differences (*p*<0.05) in each group are denoted by different alphabetical letters.

Fig 11 summarizes the bactericidal activity of photo-irradiated samples. Under the condition without the LED-light irradiation, 0.25 mg total polyphenol/ml of GPE, 0.25 mg/ml of GSE, and 0.25 mg/ml of (+)-catechin kept under a light-shielding condition for 10 min showed almost no bactericidal activity in comparison with that of the corresponding pure water group. LED-light irradiation alone showed somewhat bactericidal activity. That is, LED-light irradiation of pure water for 10 min showed approximately 1 log reduction of viable bacterial count (CFU/ml) when compared with the pure water group without LED-light irradiation. LED-light irradiation of GPE for 10 min significantly killed the bacteria in a concentration dependent manner, and 0.063 and 0.25 mg total polyphenol/ml of GPE achieved 1 and 2 log reduction of viable bacterial count, respectively, compared with that of photo-irradiated pure water group. Photo-irradiated GPE and (+)-catechin also showed bactericidal effect in a concentration dependent manner, but their effects did not exceed that of GPE.

### Scavenging activity against •OH generated by a Fenton reaction

**Fig 12:**
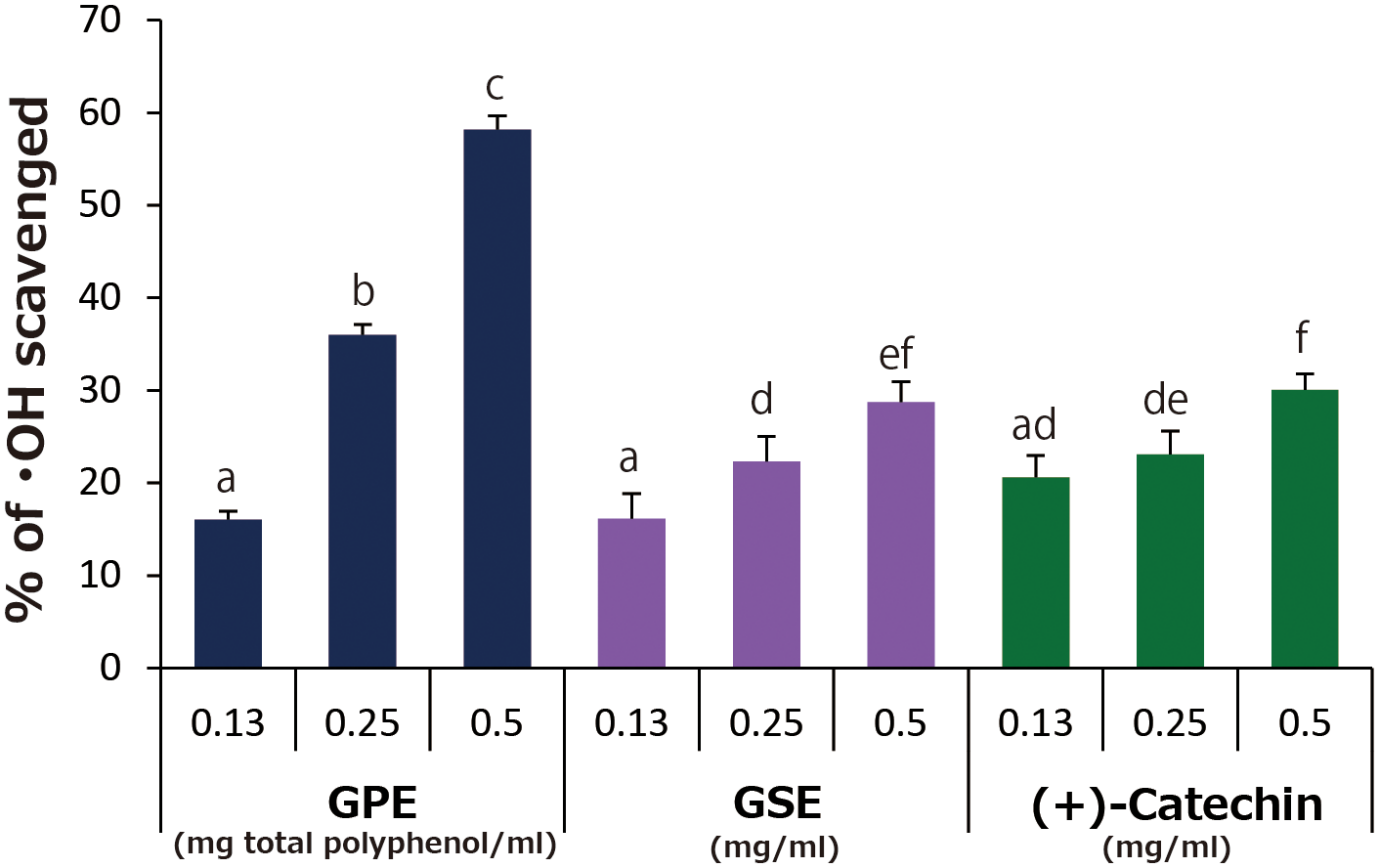
Scavenging activity of GPE, GSE, and (+)-catechin against •OH generated by a Fenton reaction. Each value represents the mean with standard deviation (n = 3). Significant differences (*p*<0.05) in each group are denoted by different alphabetical letters.

Fig 12 summarizes the scavenging activity of each sample against •OH generated by a Fenton reaction. GPE showed a concentration dependent scavenging activity, and 58, 36, and 16% of •OH were scavenged by 0.5, 0.25, and 0.13 mg total polyphenol/ml of GPE, respectively. Although GSE and (+)-catechin also showed scavenging activity, only around 30% of •OH was scavenged by the two even at the concentration of 0.5 mg/ml.

## Discussion

LC-ESI-MS analysis revealed that polyphenolic compounds including catechin monomers, dimers, trimers, and polyphenolic glucosides were contained in MeOH soluble fraction of GPE (Figs 1 and 2). Catechin dimers and trimers which correspond to m/z 579 and 867, respectively, are also known as proanthocyanidins. Since proanthocyanidins are noteworthy for their potent antioxidative potential [11] and both antioxidant and prooxidant activities likely derive from the oxidation of phenolic hydroxyl groups, it is expected that their ability to generate ROS upon photo-irradiation would be also potent. Since MeOH insoluble fraction was not suitable for MS analysis because of apprehensiveness of contamination in the ionization chamber, chemical composition of the fraction could not be analyzed. Instead, a spectrophotometric analysis showed that total polyphenol content of MeOH soluble fraction was much higher than that of MeOH insoluble fraction (Fig 3), suggesting that MeOH soluble fraction would be a major contributor for prooxidative potential of GPE. Indeed, both H_2_O_2_ and •OH generation in photo-irradiated MeOH soluble fraction were much higher than those in photo-irradiated MeOH insoluble fraction (Figs 4 and 5). Nonetheless, although total polyphenol concentration in MeOH insoluble fraction was about 1/4 of that in MeOH soluble fraction, H_2_O_2_ yields in MeOH insoluble fraction were 1/2 to 2/3 of those in MeOH soluble fraction, suggesting that polyphenols in MeOH insoluble fraction could have an ability to exert potent prooxidant activity. A further analysis for chemical composition MeOH insoluble fraction will be performed in the future. Taken these into consideration, it is suggested that polyphenolic compounds in GPE would be pivotal players as we hypothesized.

In comparison with GSE that is an authentic polyphenol product and (+)-catechin, prooxidative profiles of GPE were similar to those of GSE and (+)-catechin. That is, 1) •OH generation expressed as DMPO-OH concentration during 1 min of photo-irradiation reached a plateau at a certain concentration in all three samples tested (Fig 6), 2) in the experiment where the effect of photo-irradiation time on DMPO-OH generation was examined, DMPO-OH levels in all the samples reached a plateau at around 1 min of irradiation (Fig 7), 3) in the experiment where the effect of prior-irradiation time on the DMPO-OH generation was examined, the DMPO-OH levels reduced by only 30% in GPE and GSE after 2 hr of prior-irradiation, and the level unchanged in (+)-catechin after 2 hr (Fig 8). These results indicate that although •OH was continuously generated in all the samples at least up to 2 hr, resultant DMPO-OH did not increase after around 1 min of photo-irradiation. To explain the discrepancy between •OH and DMPO-OH generation, the decay of DMPO-OH was examined (Fig 9). Since DMPO-OH generated by photo-irradiation was relatively stable at least for 10 min regardless of the initial levels, the decay of DMPO-OH was not involved in the mechanism by which DMPO-OH level reached a plateau at around 1 min of photo-irradiation. Under the three conditions expressed as [D(+)L(+)][D(+)L(-)], [D(+)L(+)][D(+)L(+)], and [D(-)L(+)][D(+)L(+)] in which [D(+)L(+)], [D(+)L(-)], [D(-)L(+)] indicate LED-light irradiation with DMPO for 1 min, and no LED-light irradiation with DMPO for 1 min, and LED-light irradiation without DMPO for 1 min, respectively, DMPO-levels in all the three samples almost unchanged (Fig 10), suggesting that DMPO-OH would be degraded by newly formed •OH and/or be reduced to a cyclic hydroxyl amine by electrons and protons derived from photo-oxidized polyphenols. In the latter case, the cyclic hydroxylamine is ESR silent [13]. In Fig 7 for instance, this would have reduced the level of DMPO-OH in GPE from around 2 μM to 1.3 μM after photo-irradiation for 10 min.

Bactericidal activity of photo-irradiated GPE, GSE, and (+)-catechin seemed to well reflect their prooxidative potentials in terms of •OH generation. That is, bactericidal effect of photo-irradiated samples was in the order of GPE > GSE ≥ (+)-catechin (Fig 11), which was in accordance with •OH generation by photo-irradiated samples as shown in Fig 6.

Although polyphenols are noteworthy for their antioxidant activity, attention has recently been paid to their prooxidative potential. For instance, it was reported that an important anticancer and antibacterial mechanisms of polyphenols is mediated through ROS generation, which is a characteristic feature of prooxidative properties of polyphenolic compounds, leading to cancer and bacterial cell death [14,15]. Based on these findings, we hypothesized that photo-oxidation of phenolic hydroxyl group of polyphenolic compounds results in reduction of oxygen molecule to form H_2_O_2_ which in turn is photolyzed to generate •OH [4,6,7]. Since antioxidant activity of polyphenolic compounds is also mediated by the auto-oxidation of phenolic hydroxyl group [16,17], prooxidative potential and antioxidative potential of polyphenolic compounds seem to like two sides of a coin. Indeed, as was the case with •OH generation by photo-irradiated samples, scavenging activity of GPE against •OH generated by the Fenton reaction was higher than that of GSE and (+)-catechin (Fig 12). This would be attributable to their antioxidant activity. Getting back to prooxidative potential, as shown in Fig 6, a slight decrease in the DMPO-OH concentration was found in GPE with 0.5 mg/ml of total polyphenol, and no increases in 2.0 mg/ml of GSE, and 2.0 mg/ml of (+)-catechin. When the concentration is over 0.25 mg/ml total polyphenol in GPE, for instance, the antioxidant activity might prevail against •OH generation of photo-irradiated GPE resulting in the decreased or the static yield. Indeed, in our previous study in which ROS generation was examined in photo-irradiated proanthocyanidin [7], a bell-shaped yield was obtained for •OH.

In the present study, prooxidative properties of only one grape variety, Niagara, were examined. Since Niagara is a grape variety for white wine, it was of our interest to examine a grape variety for red wine. Thus, we conducted additional experiments in which grape pomace of a grape variety Zweigelt that was subjected to a vinification process of red wine was used. In the experiments, total polyphenol concentration of Zweigelt GPE was adjusted to 0.5 mg/ml, and •OH and H_2_O_2_ generation were compared with those of Niagara GPE with 0.5 mg/ml total polyphenol. As a result, •OH and H_2_O_2_ generation from photo-irradiated ZGPE were lower than those from photo-irradiated NGPE by approximately 60 and 50%, respectively (S1 Fig), suggesting that difference in variety and harvest conditions of grapes would affect prooxidative potential. To confirm this, we will try to compare prooxidative potential of multiple samples in the future.

From these, photo-irradiated GPE, one of the representative waste materials or byproducts obtained from winemaking process, could be used for a novel disinfection technique, and its prooxidative potential upon photo-irradiation would be attributable to polyphenolic compounds contained in GPE. Regarding the GPE used in the present study, the prooxidant activity in terms of •OH generation upon photo-irradiation could be retained for at least a couple of hours, and more potent than that of an authentic polyphenol product, GSE, and a pure polyphenolic compound, (+)-catechin.

## Supporting Information

S1 Fig Yields of •OH (A) and H_2_O_2_ from photo-irradiated Niagara grape pomace extract (NGPE) and Zweigelt grape pomace extract (ZGPE). Total polyphenol concentration of NGPE and ZGPE were adjusted to 0.5 mg/ml. LED light was irradiated for 1 and 10 min for •OH and H_2_O_2_, respectively. Each value represents the mean with standard deviation (n = 3).
(TIF)
