# Correction: Prooxidative Potential of Photo-Irradiated Aqueous Extracts of Grape Pomace, a Recyclable Resource from Winemaking Process

**DOI:** 10.1371/journal.pone.0160794

**Published:** 2016-08-02

**Authors:** Mana Tsukada, Takuji Nakashima, Toshiaki Kamachi, Yoshimi Niwano

There are numerical errors in Table 1. Please see the correct Table 1 and its caption here.

**Table 1:** Summary table of two-way ANOVA for the prior-irradiation time effect on DMPO-OH generation.

	df	Sum of squares	Mean square	F value	P value
Sample*	2	2.017	1.008	115.450	<0.0001
Time	3	0.892	0.297	34.036	<0.0001
Sample x Time	6	0.426	0.071	8.138	<0.0001
Error	24	0.210	0.009		

*GPE, GSE, and (+)-catechin

df: degree of freedom

There is an omission in the title of Table 2. Please see the correct Table 2 and its caption here.

**Table 2:** Summary table of two-way ANOVA for the post-irradiation time effect on DMO-OH generation.

	df	Sum of squares	Mean square	F value	P value
Sample*	2	1.430	0.715	77.7652	<0.0001
Time	3	0.036	0.0125	1.3205	0.2909
Sample x Time	6	0.061	0.010	1.0992	0.3915
Error	24	0.221	0.009		

*GPE, GSE, and (+)-catechin

df: degree of freedom

## References

[1] Tsukada M, Nakashima T, Kamachi T, Niwano Y (2016) Prooxidative Potential of Photo-Irradiated Aqueous Extracts of Grape Pomace, a Recyclable Resource from Winemaking Process. PLoS ONE 11(6): e0158197 doi:10.1371/journal.pone.0158197 27341398 10.1371/journal.pone.0158197 PMC4920348

