# Supplementary figures and images for: Prooxidative Potential of Photo-Irradiated Aqueous Extracts of Grape Pomace, a Recyclable Resource from Winemaking Process
Source: PLoS One. 2016 Jun 24;11(6):e0158197. doi: 10.1371/journal.pone.0158197 (PMC4920348; doi:10.1371/journal.pone.0158197)

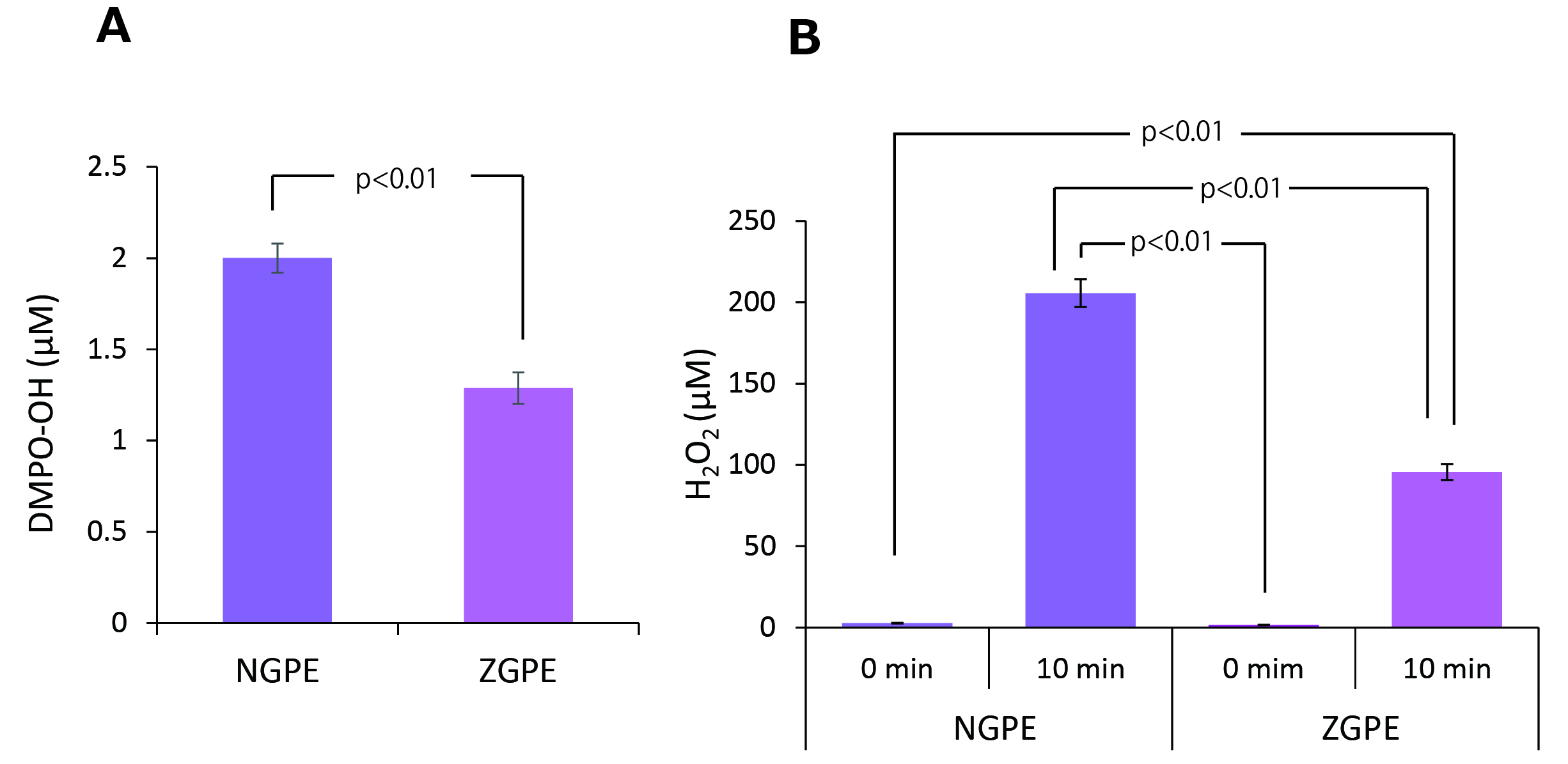

Supplement: S1 Fig — Total polyphenol concentration of NGPE and ZGPE were adjusted to 0.5 mg/ml. LED light was irradiated for 1 and 10 min for •OH and H2O2, respectively. Each value represents the mean with standard deviation (n = 3). (TIF) [file pone.0158197.s001.tif]
